# Tracking the international spread of SARS-CoV-2 lineages B.1.1.7 and B.1.351/501Y-V2

**DOI:** 10.12688/wellcomeopenres.16661.1

**Published:** 2021-05-19

**Authors:** Áine O'Toole, Verity Hill, Oliver G. Pybus, Alexander Watts, Issac I. Bogoch, Kamran Khan, Jane P. Messina, The COVID-19 Genomics UK (COG-UK) consortium, Network for Genomic Surveillance in South Africa (NGS-SA), Brazil-UK CADDE Genomic Network, Houriiyah Tegally, Richard R. Lessells, Jennifer Giandhari, Sureshnee Pillay, Kefentse Arnold Tumedi, Gape Nyepetsi, Malebogo Kebabonye, Maitshwarelo Matsheka, Madisa Mine, Sima Tokajian, Hamad Hassan, Tamara Salloum, Georgi Merhi, Jad Koweyes, Jemma L. Geoghegan, Joep de Ligt, Xiaoyun Ren, Matthew Storey, Nikki E. Freed, Chitra Pattabiraman, Pramada Prasad, Anita S. Desai, Ravi Vasanthapuram, Thomas F. Schulz, Lars Steinbrück, Tanja Stadler, Swiss Viollier Sequencing Consortium, Antonio Parisi, Angelica Bianco, Darío García de Viedma, Sergio Buenestado-Serrano, Vítor Borges, Joana Isidro, Sílvia Duarte, João Paulo Gomes, Neta S. Zuckerman, Michal Mandelboim, Orna Mor, Torsten Seemann, Alicia Arnott, Jenny Draper, Mailie Gall, William Rawlinson, Ira Deveson, Sanmarié Schlebusch, Jamie McMahon, Lex Leong, Chuan Kok Lim, Maria Chironna, Daniela Loconsole, Antonin Bal, Laurence Josset, Edward Holmes, Kirsten St. George, Erica Lasek-Nesselquist, Reina S. Sikkema, Bas Oude Munnink, Marion Koopmans, Mia Brytting, V. Sudha rani, S. Pavani, Teemu Smura, Albert Heim, Satu Kurkela, Massab Umair, Muhammad Salman, Barbara Bartolini, Martina Rueca, Christian Drosten, Thorsten Wolff, Olin Silander, Dirk Eggink, Chantal Reusken, Harry Vennema, Aekyung Park, Christine Carrington, Nikita Sahadeo, Michael Carr, Gabo Gonzalez, SEARCH Alliance San Diego, National Virus Reference Laboratory, SeqCOVID-Spain, Danish Covid-19 Genome Consortium (DCGC), Communicable Diseases Genomic Network (CDGN), Dutch National SARS-CoV-2 surveillance program, Division of Emerging Infectious Diseases (KDCA), Tulio de Oliveira, Nuno Faria, Andrew Rambaut, Moritz U. G. Kraemer

**Affiliations:** 1 Institute of Evolutionary Biology, University of Edinburgh, Edinburgh, UK; 2 Department of Zoology, University of Oxford, Oxford, UK; 3 Li Ka Shing Knowledge Institute, St. Michael's Hospital, Toronto, Canada; 4 BlueDot, Toronto, Canada; 5 Department of Medicine, University of Toronto, Toronto, Canada; 6 Divisions of General Internal Medicine and Infectious Diseases, University Health Network, Toronto, Canada; 7 Department of Geography, University of Oxford, Oxford, UK; 8 KwaZulu-Natal Research Innovation and Sequencing Platform (KRISP), Nelson R Mandela School of Medicine, University of KwaZulu-Natal, Durban, South Africa; 9 Botswana Institute for Technology Research and Innovation, Gaborone, Botswana; 10 National Health Laboratory, Gaborone, Botswana; 11 Ministry of Health and Wellness, Gaborone, Botswana; 12 Department of Natural Sciences, Lebanese American University, Beirut, Lebanon; 13 Faculty of Public Health, Lebanese University, Beirut, Lebanon; 14 Department of Microbiology and Immunology, University of Otago, Dunedin, New Zealand; 15 Institute of Environmental Science and Research, Wellington, New Zealand; 16 School of Natural and Computational Sciences, Massey University, Auckland, New Zealand; 17 Department of Neurovirology, National Institute of Mental Health and Neurosciences, Bengaluru, India; 18 Institute of Virology, Hannover Medical School, Hannover, Germany; 19 Department of Biosystems Science and Engineering, ETH Zürich, Zurich, Switzerland; 20 Istituto Zooprofilattico sperimentale della Puglia e della Basilicata, Puglia, Italy; 21 Hospital General Universitario Gregorio Marañón; Instituto de Investigación Sanitaria Gregorio Marañón, Madrid, Spain; 22 CIBER Enfermedades Respiratorias CIBERES, Madrid, Spain; 23 Bioinformatics Unit, Department of Infectious Diseases, National Institute of Health Doutor Ricardo Jorge (INSA), Lisbon, Portugal; 24 Innovation and Technology Unit, Department of Human Genetics, National Institute of Health Doutor Ricardo Jorge (INSA), Lisbon, Portugal; 25 Central Virology Laboratory, Israel Ministry of Health, Sheba Medical Center, Ramat Gan, Israel; 26 Microbiological Diagnostic Unit Public Health Laboratory, Department of Microbiology & Immunology, University of Melbourne at the Peter Doherty Institute for Infection & Immunity, Melbourne, Australia; 27 New South Wales Health Pathology - Institute of Clinical Pathology and Medical Research, Sydney, Australia; 28 New South Wales Health Pathology Randwick, Prince of Wales Hospital, Sydney, Australia; 29 Kinghorn Centre for Clinical Genomics, Sydney, Australia; 30 Queensland Reference Centre for Microbial and Public Health Genomics, Forensic and Scientific Services, Health Support Queensland, Queensland Health South Australia Pathology, Adelaide, Australia; 31 South Australia Pathology, Adelaide, Australia; 32 Department of Biomedical Sciences and Human Oncology, University of Bari, Bari, Italy; 33 Centre National de Référence des virus des infections respiratoires, Hospices Civils de Lyon, Lyon, France; 34 University of Sydney, Sydney, Australia; 35 Wadsworth Center, New York State Department of Health, Albany, New York, USA; 36 ErasmusMC, Department of Viroscience, WHO collaborating centre for arbovirus and viral hemorrhagic fever Reference and Research, Rotterdam, The Netherlands; 37 The Public Health Agency of Sweden, Department of Microbiology, Solna, Sweden; 38 Upgraded Department of Microbiology, Osmania Medical College, Hyderabad, Telangana, India; 39 Department of Virology, University of Helsinki, Helsinki, Finland; 40 HUS Diagnostic Center, HUSLAB, Clinical Microbiology, University of Helsinki and Helsinki University Hospital, Helsinki, Finland; 41 Department of Virology, National Institute of Health, Islamabad, Pakistan; 42 National Institute for Infectious Diseases "L. Spallanzani", Rome, Italy; 43 Institute for Virology, Charité Universitätsmedizin, Berlin, Germany; 44 Robert Koch-Institut, , Head, Unit 17, Influenza and other Respiratory Viruses, Seestr. 10, Berlin, Germany; 45 WHO COVID-19 reference laboratory, Centre for Infectious Disease Control-National Institute for Public Health and the Environment, Bilthoven, The Netherlands; 46 Division of Emerging Infectious Diseases, Bureau of Infectious Disease Diagnosis Control, Korea Disease Control and Prevention Agency, Cheongju-si, Chungcheongbuk-do, South Korea; 47 University of the West Indies, St. Augustine, Trinidad and Tobago; 48 National Virus Reference Laboratory, University College Dublin, Dublin, Ireland; 49 Imperial College London, London, UK

**Keywords:** genomic surveillance, air travel, SARS-CoV-2, genomics, genome sequencing, virus, surveillance, pandemic, B.1.1.7, B.1.351, N501Y, coronavirus, sequencing, genomic epidemiology

## Abstract

Late in 2020, two genetically-distinct clusters of severe acute respiratory syndrome coronavirus 2 (SARS-CoV-2) with mutations of biological concern were reported, one in the United Kingdom and one in South Africa. Using a combination of data from routine surveillance, genomic sequencing and international travel we track the international dispersal of lineages B.1.1.7 and B.1.351 (variant 501Y-V2). We account for potential biases in genomic surveillance efforts by including passenger volumes from location of where the lineage was first reported, London and South Africa respectively. Using the software tool grinch (global report investigating novel coronavirus haplotypes), we track the international spread of lineages of concern with automated daily reports, Further, we have built a custom tracking website (cov-lineages.org/global_report.html) which hosts this daily report and will continue to include novel SARS-CoV-2 lineages of concern as they are detected.

## Introduction

In December 2020, routine genomic surveillance in the United Kingdom (UK) ^1^ reported a new and genetically distinct phylogenetic cluster of severe acute respiratory syndrome coronavirus 2 (SARS-CoV-2) (variant VOC202012/01, lineage B.1.1.7). Preliminary analysis suggests that this lineage carries an unusually large number of genetic changes ^2^. The earliest known cases of B.1.1.7 were sampled in southern England in late September 2020, and by December the lineage had spread to most UK regions and was growing rapidly ^3^. In October 2020, a separate SARS-CoV-2 cluster (variant 501Y.V2, lineage B.1.351), which carried a different constellation of genetic changes, was detected by the Network for Genomic Surveillance in South Africa ^4, 5^. Both lineages carry mutations, especially in the virus spike protein, that may affect virus function, and both appear to have grown rapidly in relative frequency since their discovery. Early analyses of the spatial spread of SARS-CoV-2 highlights the potential for rapid virus dissemination through national and international travel ^6, 7^. Therefore continued genomic monitoring of lineages of concern is required.

## Methods

To better characterise the international distribution of lineages B.1.1.7 and B.1.351 we collated SARS-CoV-2 sequences from GISAID ^8, 9^ and assigned lineages using pangolin (v2.1.6, https://github.com/cov-lineages/pangolin), which implements the nomenclature scheme described in Rambaut *et al.*, ^10^. Genomes are assigned lineage B.1.1.7 if they exhibit at least 5 of the 17 mutations inferred to have arisen on the phylogenetic branch immediately ancestral to the cluster ( Table 1) ^2^; or to B.1.351 if they exhibit at least 5 of 9 lineage-associated mutations ( Table 1) ^5^. Lineage count and frequency data have been calculated daily using grinch. Using Air Transport Association (IATA) travel data from October 2020, available through bluedot.global, we aggregated and collated the passenger volumes from international airports in London and South Africa to international destinations on same booking. Destinations with more than 5,000 passengers from London and more than 300 passengers from South Africa during the month of October are displayed on the cov-lineages.org website and in the underlying data for this publication ^11^. grinch, with custom python modules that make use of geopandas v0.9, matplotlib v3.2 and seaborn v0.10, combines this information and produces reports with descriptive tables and figures that can be found at https://cov-lineages.org/global_report.html.

**Table 1.** Defining mutations for lineages of interest.

Lineage	Defining mutations
B.1.1.7	orf1ab:T1001I; orf1ab:A1708D; orf1ab:I2230T; del:11288:9; del:21765:6; del:21991:3; S:N501Y; S:A570D; S:P681H; S:T716I; S:S982A; S:D1118H; Orf8:Q27*; Orf8:R52I; Orf8:Y73C; N:D3L; N: S235F
B.1.351/501Y-V2	E:P71L; N:T205I; orf1a:K1655N; S:D80A; S:D215G; S:K417N; S:E484K; S:N501Y; S:E484K

### Implementation

All of the code underlying this daily lineage tracking web-report can be found at GitHub and Zenodo ^12^. grinch is a python-based tool, the analysis pipeline of which is built on a snakemake backbone ^13^. Every 24 hours a scheduled cron ^14^ task runs on our local servers. We download the latest data from GISAID and deduplicate based on sequence names. The sequences are assigned their most likely lineage using pangolin’s latest version and model files. All processed metadata is available and maintained on the cov-lineages.org GitHub repository. To run grinch, the user must have access to a GISAID direct download key and a password and provide these within a configuration file for use. The command used to run grinch is grinch -i grinch_config.yaml, using the config file provided at doi: 10.5281/zenodo.4640379 ^15^.

### Operation

Most users will not run grinch themselves, instead all information and useful descriptive figures are provided daily on the web report. Users can navigate to cov-lineages.org in a web browser of choice to view the latest daily report.

## Results and discussion

**Figure 1.**
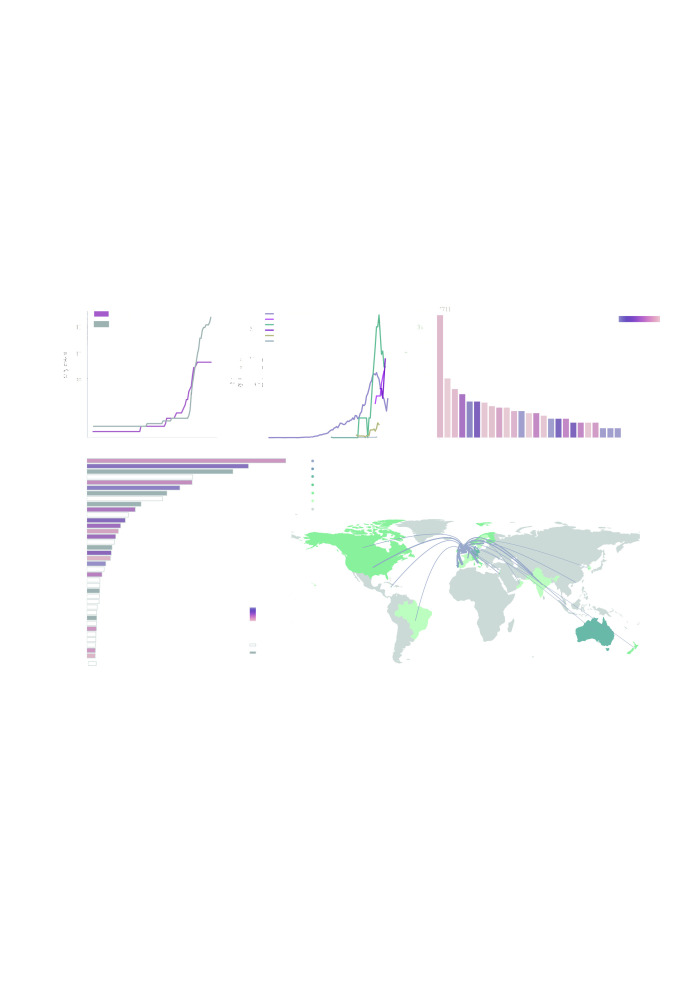
**a**) The cumulative number of countries with reports of lineage B.1.1.7 (grey line) and cumulative number of genomes of B.1.1.7 deposited in GISAID. **b**) Rolling seven-day average of the proportion of B.1.1.7 genomes in countries with more than ten sequences of the variant, and with more than ten days between the first B.1.1.7 sequence and the most recent one compared to all sampled genomes in that country. **c**) Number of sequences (log10) per country. Colour indicates the proportion of sequences that are classified as lineage B.1.1.7. **d**) Number of air travellers from major international London airports (Heathrow, Gatwick, Luton, City, Stansted, Southend) during October 2020. Colour indicates the number of sampled genomes of lineage B.1.1.7. **e**) Map of international flights from major international London airports to countries with B.1.1.7 sequences. Colours indicate the date of earliest detection of B.1.1.7. in each country. The width of the lines indicates the number of flights. International Air Transport Association data used here account for ~90% of passenger travel itineraries on commercial flights, excluding transportation via unscheduled charter flights (the remainder is modelled using market intelligence). Data shown represents origin-destination journeys during October 2020. Routes to countries that have not yet detected B.1.1.7 and deposited data on GISAID are not included.

As of 7th Jan 2021, 45 countries had reported the presence of B.1.1.7 and 13 countries had reported B.1.351/501Y.V2. B.1.1.7 and B.1.351 genome sequences were available for 28 and 8 countries, respectively ( Figure 1a, b, c) ^11^. Although some countries report increases in the relative frequency of B.1.1.7, genome sequencing efforts vary considerably. Potential targeting of sequencing towards travelers from the UK could bias frequency estimates upwards ( Figure 1b, c) and differing genome sharing policies and delays may also skew reporting estimates. The time between the initial collection date of a new variant sample in a country and the first availability of a corresponding virus genome on GISAID was, on average, 12 days (range 1–71).

The number of B.1.1.7 and B.1.351/501Y.V2 genome sequences reported in each country is a consequence of (i) the intensity of local genomic surveillance; (ii) the level of concern about new variant introductions; (iii) the volume of international travel among affected countries, and (iv) the amount of local transmission following the introduction of lineage from elsewhere. To explore these factors, we analysed the most recent available International Air Transport Association (IATA) travel data (October 2020). We collated the total number of origin-to-destination air journeys between major London international airports and each country. The calculation was repeated for journeys originating in all international South African airports. We focussed on London and South Africa as they are the locations with the first reports and highest reported prevalence of lineages B.1.1.7 and B.1.351 respectively ^2, 5^. However, due to low SARS-CoV-2 genomic surveillance in many locations, we cannot reject the hypotheses that these lineages initially originated elsewhere. Figure 1d shows destinations receiving

**Figure 2.**
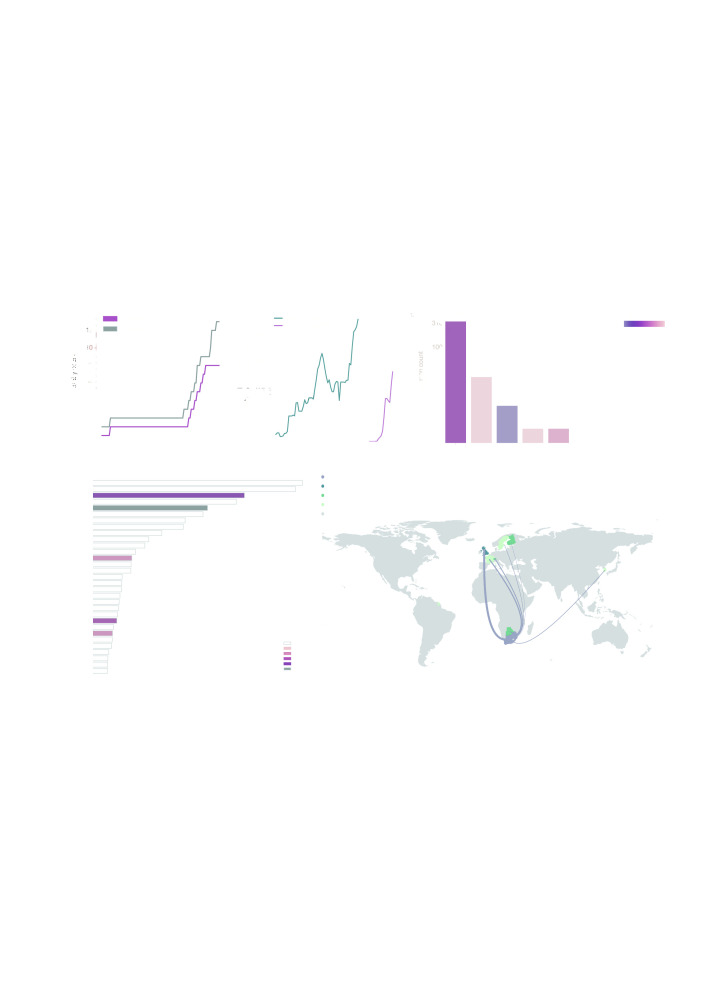
**a**) Shows the cumulative number of countries with reports of lineage B.1.351 (black line) and cumulative number of genomes of B.1.351 deposited in GISAID. **b**) Rolling seven-day average of the proportion of B.1.1.7 genomes in countries with more than ten sequences of the variant, and with more than ten days between the first B.1.1.7 sequence and the most recent one compared to all sampled genomes in that country. **c)** Number of sequences (log10) per country. Colour indicates the proportion of sequences that are classified as lineage B.1.351 **d**) Number of air travellers from South Africa during October 2020. Colour indicates the number of sampled genomes of lineage B.1.351. **e**) Map of international flights to countries with B.1.351 sequences. Colours indicate the date of earliest detection of B.1.351 in each country. The width of the lines indicates the number of flights. International Air Transport Association data used here account for ~90% of passenger travel itineraries on commercial flights, excluding transportation via unscheduled charter flights (the remainder is modelled using market intelligence). Data shown represents origin-destination journeys during October 2020. Routes to countries that have not yet detected B.1.351 and deposited data on GISAID are not included.

>5,000 travellers in October 2020 from the UK ( Figure 2 shows destinations receiving

>300 travellers from South Africa).

Of the countries that receive >5,000 travellers from London, 16 have sequenced B.1.1.7. Of the 45 countries that have identified B.1.1.7 (32 in travellers and 13 with local onward transmission), only 6 perform real-time routine genomic surveillance (Denmark, UK, Iceland, The Netherlands, Australia, Sweden), 3 have prioritised sequencing based on S-gene target failure tests ^16^, 30 primarily targeted sequencing towards arriving travellers from the UK, and there was no information available for 10 (details at https://github.com/cov-lineages/lineages-website/blob/master/_data/). Of the 13 countries that have identified B.1.351 (four with local onward transmission including South Africa), 4 perform routine sequencing (South Africa, UK, Botswana, Australia), 6 target sequencing of travellers, and there was no information available for 3. Consequently, the number of sequences reported does not correlate with flight numbers, but rather reflects current genomic surveillance effort. For example, in September, the UK sequenced ~13% of its reported cases and Denmark sequenced ~21%. In comparison, Israel sequenced ~0.002% of its cases during the same period ^17, 18^.

Our study has several limitations. The passenger flight data do not include recent changes to holiday travel, and recent restrictions on travel from the UK and South Africa is not reflected in the mobility data. Further, flight data may not accurately reflect the final destination if multiple tickets are purchased.

The discovery and rapid spread of B.1.1.7 and B.1.351/501Y.V2 highlights the importance of real-time and open data for tracking the spread of SARS-CoV-2 and for informing future public health interventions and travel advice.

## Data availability

### Underlying data

Zenodo: Accession IDs included in publication Tracking the international spread of SARS-CoV-2 lineages B.1.1.7 and B.1.351/501Y-V2. https://doi.org/10.5281/zenodo.4642401 ^9^. 

This project contains the following underlying data:

- Accession IDs of B.1.1.7 and B.1.351 genome sequences included in report up until January 7 ^th^, 2021. All accession IDs link to data on the GISAID repository, http://doi.org/10.17616/R3Q59F. These data are available under the terms of the GISAID EpiFlu™ Database Access Agreement.

Zenodo: cov-lineages.org website. https://doi.org/10.5281/zenodo.4640140 ^11^.

This project contains the following underlying data:

- Website data archived at time of publication

Data are available under the terms of the Creative Commons Attribution 4.0 International license (CC-BY 4.0).

### Extended data

Zenodo: Supplementary materials with group affiliations for Tracking the international spread of SARS-CoV-2 lineages B.1.1.7 and B.1.351/501Y-V2. https://doi.org/10.5281/zenodo.4704471 ^19^.

This project contains the following extended data:

- Supplementary materials with group authorship affiliations and full acknowledgements.

Data are available under the terms of the Creative Commons Attribution 4.0 International license (CC-BY 4.0).

## Software availability

- **Software available from:** https://cov-lineages.org/global_report.html
- **Source code available from:** https://github.com/cov-lineages/grinch
- **Archived source code at time of publication:** https://doi.org/10.5281/zenodo.4640037 ^12^; https://doi.org/10.5281/zenodo.4640379 ^15^
- **Licenses:** GNU General Public License v3.0; Creative Commons Attribution 4.0 International license (CC-BY 4.0).
