# Supplementary material for: Tracking the international spread of SARS-CoV-2 lineages B.1.1.7 and B.1.351/501Y-V2 with grinch
Source: Wellcome Open Res. 2021 Sep 17;6:121. Originally published 2021 May 19. [Version 2] doi: 10.12688/wellcomeopenres.16661.2 (PMC8176267; doi:10.12688/wellcomeopenres.16661.2)
Supplement: Peer review history

# Peer review: Reviewer response for version 1

**DOI:** 10.21956/wellcomeopenres.18372.r43967

**Published:** 2021-06-10

**Authors:** George Githinji

**Affiliations:** 1 Epidemiology and Demography Department, Kenya Medical Research Institute (KEMRI) - Wellcome Trust Research Programme, Kilifi, Kenya

The article by O’Toole 2021 *et al.* describes a bioinformatics tool for the analysis of SARS-CoV-2 sequence data. The article is concise, and the relevant details have been considered. For example, the software and source code is available and well documented. The tool has shown great utility in public health based on its application in tracking and describing two SARS-CoV-2 variants of global concern.

 Some minor comments below:

1. The transition from an article of public health importance to a software tool is abrupt. I think a paragraph or a link aimed at orientating the audience would be useful.
2. It would be useful to outline the special niche that the tool occupies or the gaps it fills relative to similar utilities and webpages such as covariants.org and outbreak.info.
3. The readme file at https://github.com/cov-lineages/grinch lacks full installation documentation. An introductory paragraph of the tool and its utility would also be useful. The scripts directory could be better organised by separating the snakemake files from the regular python files. I would image a workflows dir and scripts dir

Are the conclusions about the tool and its performance adequately supported by the findings presented in the article?

Yes

Is the rationale for developing the new software tool clearly explained?

Yes

Is the description of the software tool technically sound?

Yes

Are sufficient details of the code, methods and analysis (if applicable) provided to allow replication of the software development and its use by others?

Yes

Is sufficient information provided to allow interpretation of the expected output datasets and any results generated using the tool?

Yes

Reviewer Expertise:

bioinformatics, molecular epidemiology

I confirm that I have read this submission and believe that I have an appropriate level of expertise to confirm that it is of an acceptable scientific standard.

# Response

**Published:** 2021-09-10

**Authors:** Áine Niamh O'Toole

Thank you for your review and time, and apologies for taking so long to respond!

**The transition from an article of public health importance to a software tool is abrupt. I think a paragraph or a link aimed at orientating the audience would be useful.**

 We have added in a paragraph that bridges the public health information and the resource information. 

**It would be useful to outline the special niche that the tool occupies or the gaps it fills relative to similar utilities and webpages such as covariants.org and outbreak.info.**

 The linking paragraph also discusses other resources such as outbreak.info and covariants.org- we now also provide a non-exhaustive list of a several other resources on the cov-lineages.org website  

 ​​​​​​​

**The readme file at https://github.com/cov-lineages/grinch lacks full installation documentation. An introductory paragraph of the tool and its utility would also be useful. The scripts directory could be better organised by separating the snakemake files from the regular python files. I would image a workflows dir and scripts dir**

 We have updated the readme on the grinch repository and updated usage in that it can be run in full analysis pipeline mode or in report only mode. We have also added in a brief description of the grinch setup and where the macro data is hosted.

# Peer review: Reviewer response for version 1

**DOI:** 10.21956/wellcomeopenres.18372.r43964

**Published:** 2021-06-03

**Authors:** Anderson F. Brito

**Affiliations:** 1 Department of Epidemiology of Microbial Diseases, Yale School of Public Health, New Haven, CT, USA

Thank you for developing these tools for daily tracking of SARS-CoV-2 lineages.

**Some comments about the tool and its functionalities:**

- In the manuscript, the authors mention that users with access to GISAID direct download could run this pipeline locally, and generate their own reports. Can this tool be adapted to display genomic results for tracking national spread, or even state-level spread of lineages?
- If this pipeline is intended to be constantly executed locally by users, it would be helpful to provide more information about how to install and run the pipeline, including reference to example input and output files.  I have tried to run the pipeline using my GISAID data provision credentials, but that was not successful, as I ran into errors for which I could not find a solution online (GitHub and Zenodo).
- About the online reports, increasing the font size in the plots being displayed (bar, curves, etc) would make labels and legends more intelligible, and improving the readability of their content.
- About the flight data, why only flight counts from October are shown? Are these data only used for tracking the potential spread in early stages of viral emergence, or do you see other uses for such data?

**Concerning the manuscript, a few minor points:**

- The colour gradient in the legend of Figure 1 is incomplete and does not go from 1 to 76. I think it must be just a formatting issue.
- How was the "reported" cases shown in Figures 1 and 2 detected? By differential PCR? I know that applies to B.1.1.7, but what about B.1.351?
- The legend in Figure 2 refers to "B.1.1.7" sequences, while the figure shows "B.1.351" sequences. It must be a typo.

Are the conclusions about the tool and its performance adequately supported by the findings presented in the article?

Yes

Is the rationale for developing the new software tool clearly explained?

Yes

Is the description of the software tool technically sound?

Partly

Are sufficient details of the code, methods and analysis (if applicable) provided to allow replication of the software development and its use by others?

Partly

Is sufficient information provided to allow interpretation of the expected output datasets and any results generated using the tool?

Partly

Reviewer Expertise:

Virology, Bioinformatics, Evolution, Epidemiology.

I confirm that I have read this submission and believe that I have an appropriate level of expertise to confirm that it is of an acceptable scientific standard.

# Response

**Published:** 2021-09-10

**Authors:** Áine Niamh O'Toole

- **In the manuscript, the authors mention that users with access to GISAID direct download could run this pipeline locally, and generate their own reports. Can this tool be adapted to display genomic results for tracking national spread, or even state-level spread of lineages?**
  We’ve added in an option to grinch with the --analysis flag, that can either run the whole GISAID-processing pipeline, or just generate a set of reports from a metadata file. It currently would need resolution to remain at the country level, but we like the idea of doing a more local report and will work towards implementing something like this in the future. 
- **If this pipeline is intended to be constantly executed locally by users, it would be helpful to provide more information about how to install and run the pipeline, including reference to example input and output files.  I have tried to run the pipeline using my GISAID data provision credentials, but that was not successful, as I ran into errors for which I could not find a solution online (GitHub and Zenodo).**
  We have supplied some more information on how to install on the GitHub repository and run the pipeline, however the tool isn’t necessarily intended for users to run locally themselves as we process the data on this end and share the macro count data on GitHub.
- **About the online reports, increasing the font size in the plots being displayed (bar, curves, etc) would make labels and legends more intelligible, and improving the readability of their content.**
  We have made the axes longer to account for more countries, however are working towards re-implementing these reports in javascript so they can be interactive and more responsive to the browser size. 
- **About the flight data, why only flight counts from October are shown? Are these data only used for tracking the potential spread in early stages of viral emergence, or do you see other uses for such data?**
  October related to the date of early spread of both lineages described in the text and was due to limitations of access to data. We hope to continue to develop this resource and supply more recent dates that track over time. 

** **


**Concerning the manuscript, a few minor points:**

- **The colour gradient in the legend of Figure 1 is incomplete and does not go from 1 to 76. I think it must be just a formatting issue.**
  This was a proofs issue and we’ve hopefully rectified this now.
- **How was the "reported" cases shown in Figures 1 and 2 detected? By differential PCR? I know that applies to B.1.1.7, but what about B.1.351?**
  This was the set of manually curated media reports that we were tracking at the time.
- **The legend in Figure 2 refers to "B.1.1.7" sequences, while the figure shows "B.1.351" sequences. It must be a typo.**
  Thank you we have now fixed this in the figure.

# Response

**Published:** 2021-09-10

**Authors:** Áine Niamh O'Toole

Also- thank you for your time reviewing this manuscript (we do really appreciate it!) and apologies for taking so long to respond.

# Peer review: Reviewer response for version 1

**DOI:** 10.21956/wellcomeopenres.18372.r43966

**Published:** 2021-05-27

**Authors:** Rob Lanfear

**Affiliations:** 1 Department of Ecology and Evolution, Research School of Biology, Australian National University, Canberra, ACT, Australia

This article describes a software tool, grinch, that can be used to produce automated reports on SARS-CoV-2 lineages. The authors apply it to two lineages of concern in the article, and also highlight that the main utility of grinch is not in static one-off reports, but in regularly updated reports available at https://cov-lineages.org/global_report.html.

 The paper clearly describes the software and demonstrates its utility. I’d like to commend the authors for putting this tool and the associated website together so quickly, for maintaining both to a very high standard, for making sure that all of the work is open and reproducible, and for the huge amount of work and enormous collaborative effort that has gone into this clear and concise report.

 I have no serious reservations about the software tool or the data, analyses, or conclusions presented in the manuscript. The software is clear, open-source, sufficiently documented, and almost all of the proposed utility is presented on a clear and regularly updated website. The manuscript is clearly written, well researched, concise, and the conclusions are well justified by the analyses.

 Of course, I do have a few comments, some of which I hope might be useful in improving the paper and/or the website.

 Minor comments on the manuscript:

1. I felt there was some tension in this article about whether it’s a software note or a public health report. The title suggests the latter, but much of the article (and the article type of “Software Tool Article”) suggests the former. Most of this tension for me as a reader came from looking at the title, which has no mention of software, so I think sets up expectations that differ from what is then provided (quite reasonably) in the paper. A very simple way to address this would be to start the title with “Using grinch to track…” or to end it with “… using grinch”.
2. Similar to point 1, the abstract doesn’t actually mention ‘grinch’ or https://cov-lineages.org/global_report.html. It would seem clearer to me to incorporate in the abstract the framing that this article presents a generally applicable software tool, demonstrated on two lineages of concern.
3.  I would like to see some mention of related efforts somewhere in the report. A full detailed comparison is neither warranted nor useful here because all such websites can and should change regularly, but a couple of sentences comparing cov-lineages.org to sites like outbreak.info and covariants.org would be very useful. At a minimum, it seems useful to list the similar sites the authors are aware of, if only because the fact one can see similar patterns presented on those sites serves as a useful validation of the software presented in this paper.
4. Given the situation, this is a desirable, not a requirement, but I’d love to see some unit tests on the GitHub repo. It seems potentially important to have this when the intention is to produce daily updates for public health. (Though I note that getting the same end result from completely independent implementations on other sites is probably worth more than a lot of unit tests).
5. I struggled with Figure 1D. It wasn’t clear to me what ‘reported’ and ‘not reported’ mean. And the legend makes it really hard to figure out how colours map to counts.  
6. It’s stated that there is no correlation between the numbers of sequences and flight numbers. It would be nice to see the scatter plot for this (maybe as an inset to figure 1D?), as well as the effect size and p-value of a suitable model.
7. Following from the previous point, the explanation for the lack of a correlation with absolute numbers seems reasonable. But it still seems to me that flight numbers could correlate with the frequency of B117 at a fixed time interval from the first detected case in a given locality (thus somewhat factoring out sequencing effort in the locality). Is it possible to add this analysis?
8. Please add installation instructions to the GitHub repo

Minor comments on https://cov-lineages.org/global_report.html:

1. Figure 3 for each lineage is a map of sequence counts by region. I find the legend here completely baffling. All it states is grey=No variant (that makes sense), pink = 1 sequence (that makes sense too), and purple = ‘Max sequences’. I have no idea what to make of this. How many is ‘Max’, and how am I supposed to quantitatively interpret intermediate colours to pink and purple? It’s so obvious I’m certain there are good reasons why this isn’t already done, but it does seem like a continuous colour scale is what *should* be used here. Similar to the scale in Figure 2 (grey for no data, shades of green nicely spaced and annotated for different values of a continuous variable).
2. For the widespread lineages like B.1.1.7, there’s a lot of overplotting on Figures 4 and 5, which make the counts and the country names very difficult to read. This could be addressed by just making the figures larger.
3. The table of links to news reports is absolutely wonderful. Would it be possible to include a button here to allow users to suggest additional news links? (I assume there’s an existing mechanism for doing this, but I couldn’t find one, so if not maybe just a link to a github issue with (potentially) a pre-filled title and required information would help?)

Really minor comments about the manuscript:

1. The first use of IATA (first para of the methods) is missing “International”, i.e. it says “Using Air Transport…”.
2. The second use of IATA (second para of results) does not need to be spelled out.
3. Figure 1A seems like it is missing a second Y axis for the number of GISAID genomes reported.
4. In the PDF version and the HTML version it seems that new lines were added wherever there was a ‘>’, e.g. ‘>5,000 travellers in October’ and ‘>300 travellers from South Africa’ both (erroneously?) start on new lines.

Are the conclusions about the tool and its performance adequately supported by the findings presented in the article?

Yes

Is the rationale for developing the new software tool clearly explained?

Yes

Is the description of the software tool technically sound?

Yes

Are sufficient details of the code, methods and analysis (if applicable) provided to allow replication of the software development and its use by others?

Yes

Is sufficient information provided to allow interpretation of the expected output datasets and any results generated using the tool?

Yes

Reviewer Expertise:

Phylogenetics, molecular evolution, bioinformatics. I have a passing familiarity with SARS-CoV-2 data analysis.

I confirm that I have read this submission and believe that I have an appropriate level of expertise to confirm that it is of an acceptable scientific standard.

# Response

**Published:** 2021-09-10

**Authors:** Áine Niamh O'Toole

Thank you so much for your detailed review and our apologies for not responding sooner. All your comments were well justified and fair. We've responded to and believe have addressed your comments and concerns below. 

*I felt there was some tension in this article about whether it’s a software note or a public health report. The title suggests the latter, but much of the article (and the article type of “Software Tool Article”) suggests the former. Most of this tension for me as a reader came from looking at the title, which has no mention of software, so I think sets up expectations that differ from what is then provided (quite reasonably) in the paper. A very simple way to address this would be to start the title with “Using grinch to track…” or to end it with “… using grinch”.*

 I completely agree, it’s a very fair point! We had originally submitted the article as a public health report but this didn’t fit with the Wellcome Open Research journal remit, so resubmitted under Software Tool. I have changed the title as suggested to with with “using grinch”.

*Similar to point 1, the abstract doesn’t actually mention ‘grinch’ or https://cov-lineages.org/global_report.html. It would seem clearer to me to incorporate in the abstract the framing that this article presents a generally applicable software tool, demonstrated on two lineages of concern.*

 Our abstract and introduction now both contain reference to grinch and the reports at cov-lineages.org

*I would like to see some mention of related efforts somewhere in the report. A full detailed comparison is neither warranted nor useful here because all such websites can and should change regularly, but a couple of sentences comparing cov-lineages.org to sites like outbreak.info and covariants.org would be very useful. At a minimum, it seems useful to list the similar sites the authors are aware of, if only because the fact one can see similar patterns presented on those sites serves as a useful validation of the software presented in this paper.*

 We have added in a short paragraph about these resources and a link to a more extensive (but definitely non-exhaustive) list of resources.

*Given the situation, this is a desirable, not a requirement, but I’d love to see some unit tests on the GitHub repo. It seems potentially important to have this when the intention is to produce daily updates for public health. (Though I note that getting the same end result from completely independent implementations on other sites is probably worth more than a lot of unit tests).*

 Since publication, we’ve re-worked the back-end analysis pipeline and the GISAID data is now processed with the datapipe pipeline written by Rachel Colquhoun (https://github.com/COG-UK/datapipe). The reports and webpages are still generated with grinch, however the main data processing steps are now done with the robust datapipe pipeline.

*It’s stated that there is no correlation between the numbers of sequences and flight numbers. It would be nice to see the scatter plot for this (maybe as an inset to figure 1D?), as well as the effect size and p-value of a suitable model. Following from the previous point, the explanation for the lack of a correlation with absolute numbers seems reasonable. But it still seems to me that flight numbers could correlate with the frequency of B117 at a fixed time interval from the first detected case in a given locality (thus somewhat factoring out sequencing effort in the locality). Is it possible to add this analysis?*

 We have amended to state there is no clear relationship, rather than correlation.

*Please add installation instructions to the GitHub repo.*

 We have added updated usage and install instructions, and a description of the behaviour to the repository, at https://github.com/cov-lineages/grinch/blob/main/README.md

**Minor comments on https://cov-lineages.org/global_report.html:**

1. **Figure 3 for each lineage is a map of sequence counts by region. I find the legend here completely baffling. All it states is grey=No variant (that makes sense), pink = 1 sequence (that makes sense too), and purple = ‘Max sequences’. I have no idea what to make of this. How many is ‘Max’, and how am I supposed to quantitatively interpret intermediate colours to pink and purple? It’s so obvious I’m certain there are good reasons why this isn’t already done, but it does seem like a continuous colour scale is what should be used here. Similar to the scale in Figure 2 (grey for no data, shades of green nicely spaced and annotated for different values of a continuous variable).**
  We have since amended the legend for the report. 
2. **For the widespread lineages like B.1.1.7, there’s a lot of overplotting on Figures 4 and 5, which make the counts and the country names very difficult to read. This could be addressed by just making the figures larger.**
  We have made these figures larger, but recognise that these plots may be better displayed by having a top 20 country barchart (like in https://cov-lineages.org/lineage.html?lineage=B.1.1.7) and intend to adopt an interactive, more attractive global report in the future.
3. **The table of links to news reports is absolutely wonderful. Would it be possible to include a button here to allow users to suggest additional news links? (I assume there’s an existing mechanism for doing this, but I couldn’t find one, so if not maybe just a link to a github issue with (potentially) a pre-filled title and required information would help?)**

**          **We really like this idea and will work towards implementing it!
